# Comparison of the Performance of Different Bile Salts in Enantioselective Separation of Palonosetron Stereoisomers by Micellar Electrokinetic Chromatography

**DOI:** 10.3390/molecules27165233

**Published:** 2022-08-16

**Authors:** Shaoqiang Hu, Tao Sun, Rui Li, Dongdong Zhang, Yonghua Zhang, Zhuo Yang, Ge Feng, Xuming Guo

**Affiliations:** 1 Henan Key Laboratory of Function-Oriented Porous Materials, College of Chemistry and Chemical Engineering, Luoyang Normal University, Luoyang 471934, China; 2 School of Chemical Engineering & Pharmaceutics, Henan University of Science and Technology, Luoyang 471003, China

**Keywords:** chiral separation, micellar electrokinetic chromatography, capillary electrophoresis, palonosetron hydrochloride, bile salts

## Abstract

Bile salts are a category of natural chiral surfactants which have ever been used as the surfactant and chiral selector for the separation of many chiral compounds by micellar electrokinetic chromatography (MEKC). In our previous works, the application of sodium cholate (SC) in the separation of four stereoisomers of palonosetron (PALO) by MEKC has been studied systematically. In this work, the parameters of other bile salts, including sodium taurocholate (STC), sodium deoxycholate (SDC), and sodium taurodeoxycholate (STDC) in the separation of PALO stereoisomers by MEKC were measured and compared with SC. It was found that all of four bile salts provide chiral recognition for both pairs of enantiomers, as well as achiral selectivity for diastereomers of different degrees. The structure of steroidal ring of bile salts has a greater impact on the separation than the structure of the side chain. The varying separation results by different bile salts were elucidated based on the measured parameters. A model to describe the contributions of the mobility difference of solutes in the aqueous phase and the selectivity of micelles to the chiral and achiral separation of stereoisomers was introduced. Additionally, a new approach to measure the mobility of micelles without enough solubility for hydrophobic markers was proposed, which is necessary for the calculation of separation parameters in MEKC. Under the guidance of derived equations, the separation by SDC and STDC was significantly improved by using lower surfactant concentrations. The complete separation of four stereoisomers was achieved in less than 3.5 min by using 4.0 mM of SDC. In addition, 30.0 mM of STC also provided the complete resolution of four stereoisomers due to the balance of different separation mechanisms. Its applicability for the analysis of a small amount of enantiomeric impurities in the presence of a high concentration of the effective ingredient was validated by a real sample.

## 1. Introduction

The stereochemistry of drugs is an important issue because stereoisomers often differ in the pharmacological activity, as well as toxicology or pharmacodynamics [1,2]. This makes the separation of enantiomers a hot topic in separation science for more than four decades [3,4,5]. Various techniques for the separation of enantiomers, particularly on an analytical scale, have been developed in the fields of liquid chromatography, gas chromatography, supercritical fluid chromatography, and capillary electrophoresis (CE) [6,7,8,9,10,11]. Micellar electrokinetic chromatography (MEKC) as an important mode of CE is widely used for the separation of many compounds, including the enantiomers of chiral compounds [12,13,14]. MEKC utilizes surfactants at concentrations above the critical micelle concentration (CMC) to form micelles in a background electrolyte solution (BGE), which play the role of a pseudo-stationary phase (PSP). The separation is achieved through the partition of analytes between the PSP and aqueous phase, as well as their mobility difference in an aqueous solution [15,16,17,18].

Bile salts are natural chiral surfactants with a rigid and planar structure, which have ever been used as the surfactant and chiral selector (solely or in conjunction with another chiral additive) for the enantioseparation of many chiral compounds, including amino acids and peptides, pharmaceuticals, pesticides, and environmental pollutants [19,20,21,22,23,24]. Unlike the commonly used surfactants with a long akyl chain, e.g., cetyltrimethylammonium bromide (CTAB) or sodium dodecyl sulfate (SDS), whose amphipathicity arises from their hydrophilic head and hydrophobic tail, the amhipathicity of bile salts arises from their hydrophilic and hydrophobic faces [24,25]. They aggregate in a stepwise manner into pre-micellar aggregates, primary and secondary micelles of disc-like, as well as helical, structures with a different enantioselective effect as the concentration is increased [26,27,28]. Different bile salts are differentiated by the number and stereochemistry of hydroxyl groups in the steroidal ring, and the carbon side chain (Figure S1). 

Palonosetron hydrochloride (PALO 3aS, 2S), (3aS)-2-[(S)-1-azabi-cyclo[2.2.2]oct-3- yl]-2,3,3a,4,5,6-hexahydro-1*H*-benz[*de*]isoquinolin-1-one hydrochloride (Figure S2), is a highly selective second generation 5-HT3 receptor antagonist used for the prevention of nausea and vomiting associated with chemotherapy [29,30,31]. It contains two stereogenic centers in the molecular structure and, thus, has four stereoisomers, i.e., PALO (3aS, 2S), PALO (3aR, 2R), PALO (3aS, 2R), and PALO (3aR, 2S). Among them, only PALO (3aS, 2S) possesses pharmacological activity [32,33,34]. In our previous works, bile salt sodium cholate (SC) has been used as the surfactant and sole chiral selector for the separation of PALO stereoisomers in MEKC [35,36,37,38], following the work of Tian et al. [39]. It was found that SC micelles in a wide range of concentrations from 20 to 70 mM provide good resolution for two pairs of enantiomers, i.e., PALO (3aS, 2S)/PALO (3aR, 2R) and PALO (3aS, 2R)/PALO (3aR, 2S). However, the separation of diastereomers PALO (3aR, 2R) and PALO (3aS, 2R) is challenging due to the offset of two effects—their mobility difference in aqueous phase and the selectivity of micelles. Some approaches had to be undertaken in order to accomplish the best match between two effects and the complete separation of four stereoisomers. This includes the addition of organic solvents (methanol or butanol) or additional surfactant (SDS), fine-tuning the pH and SC concentration. The performance of other bile salts was not tested.

In order to fully understand the performance of other bile salts and find alternative approaches for the separation of PALO stereoisomers, the application of other bile salts, including sodium deoxycholate (SDC), sodium taurocholate (STC), and sodium taurodeoxycholate (STDC), for the separation of PALO stereoisomers in MEKC was verified and compared with SC in this work. The varying separation results by different bile salts were elucidated based on the measured parameters and introduced model.

## 2. Materials and Methods

### 2.1. Chemicals and Reagents

The four enantiomerically pure PALO stereoisomers were purchased from J&K Scientific Ltd. (Beijing, China). Sodium cholate (SC) was purchased from Serva Feinbiochemica (Heidelberg, Germany). Sodium deoxycholate (SDC), sodium taurocholate (STC), and sodium taurodeoxycholate (STDC) were supplied by Sigma-Aldrich (St. Louis, MO, USA). Sodium tetraborate, sodium hydroxide and hydrochloric acid were produced by Xi’an Chemical Reagent Factory (Xi’an, China). PALO injection (PALO (3aS, 2S) content 50 μg∙mL^−1^) was supplied by Chia Tai Tianqing Pharmaceutical Co., Ltd. (Jiangsu, China) and used for method validation. All chemicals used were of analytical regent grade and were used without further purification.

### 2.2. Preparation of Separation Media (BGE) and Sample Solutions

Micellar solutions for MEKC were prepared by dissolving appropriate quantities of each bile salt surfactant and sodium tetraborate buffer in distilled water to the desired volume in a flask. The solution was sonicated while covered for 15 min to form a transparent micellar solution. The pH of the prepared solutions (BGE) was adjusted to an appropriate value with 1.0 M HCl or NaOH under the monitoring of a PHS-3C pH meter (Shanghai Precision & Scientific Instrument Co., Ltd., Shanghai, China). For details about the ingredients, concentrations, and pH of BGEs, see the corresponding figure captions.

The sample solutions of enantiomerically pure standards, used in the assessment of the method, were prepared by dissolving appropriate quantities of each enantiomerically pure PALO steroisomer in the corresponding BGE. A small amount of dimethyl sulfoxide (DMSO) was added as the marker of electroosmotic flow (EOF). The spiked sample solutions, for the validation of the developed method, were prepared by adding appropriate quantities of enatiomeric impurity standards into the PALO injection to concentrations ranging from 0.5 to 5.0 μg∙mL^−1^. All solutions were filtered through a 0.45 μm filter prior to use.

### 2.3. CE Experiments

CE experiments were performed on a TH-3100 capillary electrophoresis system equipped with a UV detector (Tianhui Institute of Separation Science, Hebei, China). The detection wavelength was 214 or 254 nm, depending on the bile salt used. An uncoated fused silica capillary of id 50 μm × od 365 μm (Yongnian Ruifeng Chromatographic Device Co. Ltd., Hebei, China) was used, with a total length (*L*_tot_) of 60.0 cm and an effective length (*L*_eff_) of 50.0 cm. New capillaries were pretreated by flushing in sequence with distilled water for 5 min, 1.0 M NaOH for 10 min, and distilled water for 5 min again at 140 kPa (approximately 20 psi). Between injections, the capillary was rinsed in sequence with distilled water, 1.0 M NaOH, distilled water again, and, finally, BGE for 2 min each at 140 kPa. The capillary cartridge temperature was set at 20 °C. Injections were performed hydrodynamically at 5 kPa for 1 s to 2 s, depending on the concentration. An applied voltage of +25 kV was used for all experiments unless otherwise specified.

For details about the CE conditions, see the corresponding figure captions.

## 3. Theory and Calculation

### 3.1. Model of Chiral and Achiral Separation

In MEKC, a charged solute is partitioned between the micelles and aqueous phase; its effective mobility *μ*_eff_ is the weighted average of two forms of existence. With expression of the molar fractions of the solute distributed in two phases by the retention factor, the effective mobility of the charged solute is obtained:

(1) $$\mu_{\mathrm{eff}}=\frac{\mu_{f,\mathrm{mc}}}{1+k}+\frac{k\mu_{\mathrm{mc}}}{1+k}$$

where *μ*_f,mc_ and *μ*_mc_ are the mobility of free solute distributed in the aqueous phase of micellar solution and the mobility of micelles, respectively, and *k* is the retention factor —the ratio of the amount of solute distributed in micelles relative to that in the aqueous phase [16,36].

PALO stereoisomers are of weak bases with tertiary amino group (Figure S2). They are protonated in the pH range of MEKC separation and, hence, exist as positively charged ions in the water phase. However, the pKa of protonated ions are slightly different for the two pairs of enantiomers. According to our previous work, the pKa of protonated ions for the first pair of enantiomers, PALO (3aS, 2S) and PALO (3aR, 2R), is 9.12, while for the second pair of enantiomers, PALO (3aS, 2R) and PALO (3aR, 2S), it is 9.01 [35]. Therefore, the first pair of enantiomers has a bigger extent of ionization and, hence, a bigger mobility in the aqueous phase compared to the second pair, in the range of pH 8.0–10.0, with the biggest difference around the pKa. This contributes to the separation of diastereomers between two enantiomeric pairs, in addition to the selectivity of micelles or the retention difference to micelles.

For a given MEKC system, the mobility of micelles *μ*_mc_ can be regarded as a constant. So, the effective mobility of a PALO stereoisomer *μ*_eff_ is a function of two variables –*μ*_f,mc_ (mobility of free solute) and *k* (retention factor) depicted by Equation (1). Additionally, the difference in effective mobility between two diastereomers caused by their mobility difference Δ*μ*_f,mc_ and retention difference Δ*k* can be expressed as the sum of the products of the partial derivative and the variation of each variable, i.e.,

(2) $$\Delta \mu_{\mathrm{eff}}=\frac{\partial \mu_{\mathrm{eff}}}{\partial \mu_{f,\mathrm{mc}}}\Delta \mu_{f,\mathrm{mc}}+\frac{\partial \mu_{\mathrm{eff}}}{\partial k}\Delta k$$

To calculate the partial derivatives from Equation (1) and substitute into Equation (2), yields

(3) $$\Delta \mu_{\mathrm{eff}}=\frac{1}{1+k}\Delta \mu_{f,\mathrm{mc}}+\frac{\mu_{\mathrm{mc}}-\mu_{f,\mathrm{mc}}}{{\left(1+k\right)}^{2}}\Delta k$$

The two terms on the right-hand side of Equation (3) represent the contribution of mobility difference and retention difference, respectively, to the separation.

Since a pair of enantiomers have exactly the same pKa and, thus, the same mobility in the aqueous phase, i.e., Δ*μ*_f,mc_ = 0, Equation (3) for the chiral separation in each pair of enantiomers is reduced to:

(4) $$\Delta \mu_{\mathrm{eff}}=\frac{\mu_{\mathrm{mc}}-\mu_{f,\mathrm{mc}}}{{\left(1+k\right)}^{2}}\Delta k$$

It means that only the retention difference works in the chiral separation of two pairs of enantiomers, which is different from the achiral separation of diastereomers where both mechanisms work independently. They can be synergistic or conflict each other, depending on the elution orders determined by each mechanism.

### 3.2. Calculation of Retention Factors and Selectivities

From Equation (1) to calculate the effective mobility *μ*_eff_ of a charged solute in MEKC, the retention factor *k* can be derived as

(5) $$k=\frac{\mu_{f,\mathrm{mc}}-\mu_{\mathrm{eff}}}{\mu_{\mathrm{eff}}-\mu_{\mathrm{mc}}}$$

where *μ*_f,mc_ and *μ*_mc_ are the mobility of free PALO stereoisomer distributed in the aqueous phase of micellar solution and the mobility of bile salt micelles, respectively. The *μ*_eff_ and *μ*_mc_ were calculated from the migration times of PALO stereoisomers and bile salt micelles, respectively.

Since generally used micelle markers, e.g., 1-phenyldodecane, have not enough solubility in bile salt micelles, a new approach to measure the migration time of micelles was introduced in this study. A plug of blank buffer with the same concentration and pH as in the BGE of MEKC is injected. It forms a zone depletion of micelles in the capillary that moves in the same velocity as the micelles when the high voltage is applied and, thus, can also be used as a micelle marker. When this zone reaches the detection window, a negative peak appears which represents the migration time of micelles (Figure S3).

*μ*_f,mc_ was approximately calculated using the electrophoretic mobilities obtained under conditions of capillary zone electrophoresis (CZE) and a viscosity correction for the difference between the CZE buffer and the micellar solution:

(6) $$\mu_{f,\mathrm{mc}}=\mu_{f,\mathrm{CZE}}.\nu$$

where *μ*_f,CZE_ is the measured electrophoretic mobility of PALO stereoisomer in CZE mode with equivalent conditions to MEKC and *ν* is the correction factor [40,41]. The EOF was used as a mobility standard (MOS) for viscosity correction [41,42,43,44]:

(7) $$\nu =\frac{\mu_{\mathrm{EOF},\mathrm{mc}}}{\mu_{\mathrm{EOF},\mathrm{CZE}}}=\frac{t_{0,\mathrm{CZE}}}{t_{0,\mathrm{mc}}}$$

where *t*_0,CZE_ and *t*_0,mc_ are the migration times of the EOF marker DMSO in CZE and MEKC mode, respectively.

The ratio of retention factors of two stereoisomers is defined as the selectivity. Configurations of the two stereoisomers or enantiomers discussed were annotated as subscript, e.g., *α*_3aR,2R/3aS,2S_ = *k*_3aR,2R_/*k*_3aS,2S_. Unlike the general expression of the selectivity with the bigger retention factor at the numerator and the smaller one at the denominator, and, thus, having a value never less than the unity, here the retention factor of the front stereoisomer in the subscript was fixed at the numerator and the retention factor of the rear one was fixed at the denominator, for the convenience of comparing the selectivities of different bile salts. Therefore, calculated selectivities may have any values bigger or less than the unity depending on the relative magnitude of two retention factors.

## 4. Results and Discussion

### 4.1. Comparison of Separation Performance of Different Bile Salts

**Figure 1:**
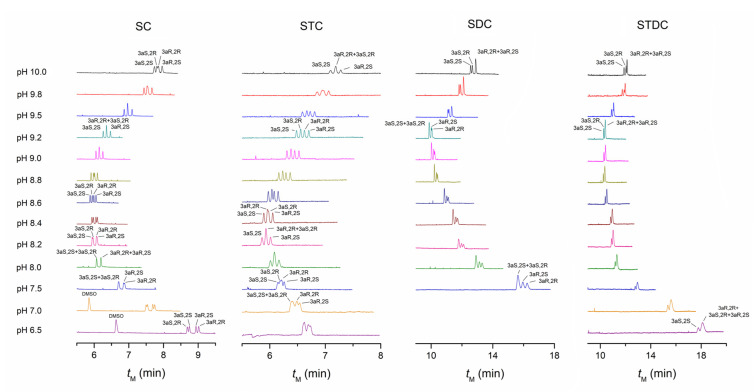
Electropherograms of PALO stereoisomers obtained with four bile salts at different pHs. Component of micellar solution is 30 mM each bile salt in 30 mM sodium tetraborate buffer. Capillary: id 50 μm, *L*_tot_ 60.0 cm, *L*_eff_ 50.0 cm. Capillary temperature: 20 °C. Detection wavelength: 214 nm for SC and SDC, 254 nm for STC and STDC. Sample concentration: 0.1 mg·mL^−1^ for each stereoisomer. Hydrodynamic injection at 5 kPa for 2 s. Applied voltage: 25 kV.

Figure 1 is the electropherograms of PALO stereoisomers at different pHs obtained with four bile salts of the same concentration of 30 mM. Among them, the result by SC was copied from our previous work [35] for the comparison with other bile salts. Additionally, we first planned to perform tests in the range of pH 6.5 to 11.5 and to calculate the retention factors of all bile salts for each stereoisomer existing in the form of both protonated ions and neutral molecules [35]. Nevertheless, PALO stereoisomers were not eluted normally, giving very broad peaks, long and not repeated migration times when STC, SDC, and STDC were used at the pH higher than 10.0 (data not given). Additionally, stereoisomers could not be eluted within 30 min when SDC was used at the pH lower than 7.5 because the solution became too sticky, probably due to the change of micelles in size or structure. Finally, the separation parameters of different bile salts at pH 9.2, the initial value of borate buffer without adjustment by acid or base, were calculated for comparison (Table 1). Here, the retention factors obtained are just pH-dependent overall values [41]—the average of protonated ions and neutral molecules. As shown in Table 1, the time windows in MEKC, *t*_0_/*t*_mc_, are roughly identical for different bile salts, except that the time window of SC is slightly small. All the bile salts tested have chiral recognition for both pairs of enantiomers, as well as selectivities for diastereomers to some extent. SC and STC, as well as SDC and STDC, with the same structure as a steroidal ring, but different structure at side chain (Figure S1), have a similar retention and selectivity to PALO stereoisomers. However, SC and SDC, as well as STC and STDC, with side chains of the same structure but steroidal rings differentiated by the hydroxyl group at C7 have rather different separation parameters. It suggests that, compared to the structural difference in the side chain, the structural difference in steroidal ring has a bigger impact on the interactions between the micelles and stereoisomers.

#### 4.1.1. The Performance of SC and STC

As shown in Table 1, the enantioselectivities of SC for two pairs of enantiomers are higher than those of STC. In addition, the enantioselectivity of SC for the first pair of enantiomers, PALO (3aS, 2S) and PALO (3aR, 2R), is slightly lower than that for the second pair, PALO (3aS, 2R) and PALO (3aR, 2S); whereas the enantioselectivities of STC for two pairs of enantiomers are largely equal. It was reflected in electropherograms that the resolutions of two pairs of enantiomers by SC are overall better than those by STC. Additionally, the resolution of the second pair of enantiomers by SC is a little higher than that of the first pair, while the resolutions of two pairs of enantiomers by STC are largely equal at pH 9.2 (Figure 1). However, they both provide enough resolution for two pairs of enantiomers. In addition to the selectivities of micelles, another mechanism, the mobility difference in the aqueous phase also contributes to the separation of PALO stereoisomers between two enantiomeric pairs, i.e., achiral separation of diastereoisomers [35,36,37,38]. Since the first pair of enantiomers, PALO (3aS, 2S) and PALO (3aR, 2R), have a bigger mobility in aqueous phase compared to the second pair, PALO (3aS, 2R) and PALO (3aR, 2S), with the biggest difference around the pKa (9.12 and 9.01 for the first pair and the second pair, respectively [35]), the peaks of the first pair of enantiomers as a whole are in front of those of the second pair in electropherograms obtained by SC and STC at pH 9.0 to 9.2.

SC gives a relatively higher selectivity of 1.054 for diastereomers PALO (3aR, 2R) and PALO (3aS, 2R), yet this effect is in conflict with the separation by mobility difference as they determine migration orders opposite each other. They cancel each other, resulting in the overlap of peaks at pH 9.2 (Figure 1). It means that the two terms on the right-hand side of Equation (3) have the equal values but opposite signs. The change in pH upwards or downwards from the value of pKa will lower the mobility difference and, thus, tip the balance between two effects, cause the resolution of two diastereomers with an elution order determined by the selectivity of SC micelles, i.e., PALO (3aS, 2R) before PALO (3aR, 2R). From another angle, this weakens the separation between two enantiomeric pairs. They approach each other, leading to the cross of their peaks and, thus, the separation of PALO (3aR, 2R) and PALO (3aS, 2R). The complete resolution of four stereoisomers can be obtained at a proper pH to achieve the best balance of different mechanisms of separation. It is pH 8.6 for SC. Like SC, the selectivity of STC for PALO (3aR, 2R) and PALO (3aS, 2R) also gives the opposite effect to the separation by mobility difference. However, its strength is not high enough to offset the latter, i.e., the absolute value of the second term is smaller than that of the first term on the right-hand side of Equation (3). As a result, the two diastereomers are separated by their mobility difference with an elution order of PALO (3aR, 2R) before PALO (3aS, 2R) determined by the mobility at pH 9.2, different from the result of SC. From another angle, relatively low enantioselectivities of STC just avoids the peak overlapping between two pairs of enantiomers and provides the complete resolution of four stereoisomers in the range of pH 8.8–9.5 around the initial value of the borate buffer. Further adjustment of the pH upwards or downwards decreases the mobility difference and, thus, the separation between two pairs of enantiomers, leading to the co-elution of PALO (3aR 2R) and PALO (3aS, 2R) even the cross of the peaks between two enantiomeric pairs, in a similar trend as SC.

#### 4.1.2. The Performance of SDC and STDC

Unlike SC and STC, the enantioselectivities of SDC and STDC for the first pair of enantiomers are evidently higher than those for the second pair; in particular, STDC gives a very low selectivity of 1.032 for PALO (3aS 2R) and PALO (3aR, 2S) (Table 1). It seems that the interaction of hydroxyl group at C7 of steroidal ring with the analyte has a great influence on the chiral recognition for the second pair of enantiomers. In addition, the retention of SDC and STDC micelles is much higher than that of SC and STC, with factors bigger than 10, probably due to their strong hydrophobic interactions with the analyte. As can be seen in Figure 1, SDC provides baseline resolutions for two pairs of enantiomers, but STDC only provides baseline resolutions for the first pair of enantiomers, at pH 9.2. The second pair of enantiomers, PALO (3aS 2R) and PALO (3aR, 2S), cannot be separated by STDC. Furthermore, the peaks of two pairs of enantiomers are close to or even superposed on each other, there is a lack of enough separation between the two pairs when SC and SDC are used. This can be ascribed to high retentions of SDC and STDC, which cause very low fractions of the analyte distributed in the aqueous phase. Therefore, the contribution of mobility difference, represented by the first term in Equation (3), that dominates the separation between two pairs of enantiomers is absent. Additionally, the separation by SDC and STDC in terms of both resolution and elution order does not change evidently with the pH, unlike the results of SC and SDC. This, too, is the effect of high retentions which remarkably reduce the contribution of mobility difference between two pairs of enantiomers. Therefore, the evident change in separation at different pHs caused by the change in mobility difference Δ*μ*_f,mc_ does not appear when SDC and STDC are used. The backward movement of the peaks of PALO (3aS 2R) and PALO (3aR, 2S) relative to the first pair of enantiomers as the pH increases from 9.2 to 10.0, when SDC is used, can be attributed to its varying selectivity for stereoisomers in the form of neutral molecules and protonated ions. The increase in pH changes their ratio and, hence, the overall selectivity.

### 4.2. Effect of SDC and STDC Concentration on the Separation

#### 4.2.1. Theoretical Consideration

As discussed above, the separation of PALO stereoisomers in MEKC relies on two mechanisms, the mobility difference of free stereoisomers in the aqueous phase and the retention difference to micelles. They both are responsible for the achiral separation of diastereomers between two enantiomeric pairs, expressed by Equation (3); whereas only the latter, the retention difference or the selectivity of micelles, works in the chiral separation in each pair of enantiomers, as expressed by Equation (4).

However, a too high retention of SDC and STDC at 30 mM (Table 1) reduces the first term on the right-hand side of Equation (3) by amplifying the denominator that represents the contribution of the mobility difference to the achiral of diastereomers. Similarly, it reduces the second term in Equation (3), as well as the term on the right-hand side of Equation (4), which represents the contribution of the retention difference to both the achiral of diastereomers and the chiral separation of enantiomers. So, a concentration of 30 mM may be inappropriate for SDC and STDC, because it gives too high retention, being unfavorable for both the chiral separation and achiral separation of PALO stereoisomers. It would improve the separation by using a lower concentration to give appropriate retention factors as those of SC and STC. Additionally, it can also shorten the separation time simultaneously. Based on above consideration, different concentrations lower than 30 mM were tested for SDC and STDC.

#### 4.2.2. Experiment Results

**Figure 2:**
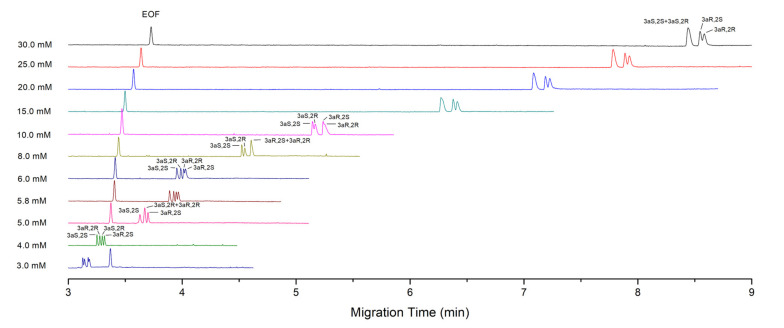
Electropherograms of PALO stereoisomers obtained with different concentrations of SDC. Component of micellar solution is SDC of annotated concentrations in 30 mM sodium tetraborate buffer of pH 9.2. Sample concentration: 0.1 mg·mL^−1^ for each stereoisomer. Hydrodynamic injection at 5 kPa, 2 s and 1 s for the SDC concentration of 30.0–10.0 mM and 8.0–3.0 mM, respectively. Detection wavelength: 214 nm. Other CE conditions are the same as in Figure 1.

Figure 2 shows the electropherograms of PALO stereoisomers obtained with different concentrations of SDC at a fixed pH of 9.2. It can be seen that the migration times of analytes are shortened remarkably with the decrease in SDC concentration. Furthermore, the peaks of the first pair of enantiomers, PALO (3aS 2S) and PALO (3aR, 2R), shift forwards gradually relative to the second pair, PALO (3aS 2R) and PALO (3aR, 2S), with the decrease in SDC concentration. It results in various migration orders of four stereoisomers at different concentrations. This can be attributed to the low retention caused by low SDC concentrations that enhances the separation between two pairs of enantiomers with different mobilities in aqueous phase. Therefore, the first pair of enantiomers with a bigger mobility shifts forwards and gradually separated with the second pair. The chiral separation in each pair of enantiomers also improves slightly as the SDC concentration is decreased from 30 mM till 8.0 mM. The complete separation of all the four stereoisomers is achieved at a SDC concentration of 4.0 mM in less than 3.5 min (Figure 2).

**Figure 3:**
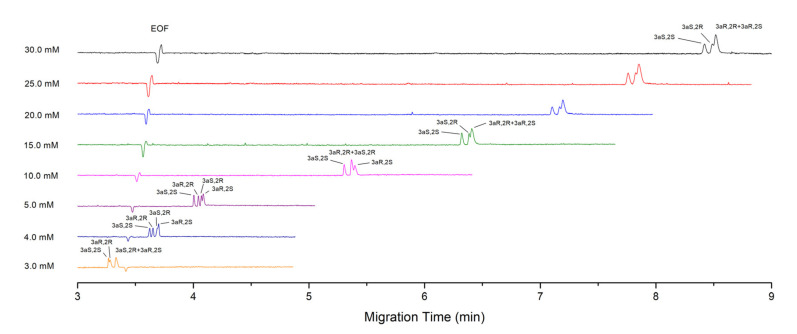
Electropherograms of PALO stereoisomers obtained with different concentrations of STDC. Component of micellar solution is STDC of annotated concentrations in 30 mM sodium tetraborate buffer of pH 9.2. Sample concentration: 0.1 mg·mL^−1^ for each stereoisomer. Hydrodynamic injection at 5 kPa, 2 s and 1 s for the STDC concentration of 30.0–10.0 mM and 5.0–3.0 mM, respectively. Detection wavelength: 254 nm. Other CE conditions are the same as in Figure 1.

Figure 3 is the electropherograms of PALO stereoisomers obtained with different concentrations of STDC at a fixed pH of 9.2. It shows the same trends as that of SDC, i.e., as the STDC concentration decreases the migration time shortens remarkably and the peaks of the first pair of enantiomers shift forwards gradually relative to the second pair, leading to the change in migration order of four stereoisomers. Additionally, the chiral separation in each pair of enantiomers improves as the surfactant concentration decreases till 10.0 mM. The best separation among four stereoisomers is obtained by 5.0 mM STDC in about 4 min, but the complete resolution of all the stereoisomers cannot be achieved, owing to low enantioselectivity of SDTC for the second pair of enantiomers, PALO (3aS 2R) and PALO (3aR, 2S) (Table 1).

In Figure 2 and Figure 3, the electropherograms corresponding to a concentration of 30 mM have equivalent resolutions to those in Figure 1, but the migration time is a little short. This is because they were made at different seasons. The increase in temperature of the uncontrolled part of capillary, as well as two vials due to the fact that the increase in room temperature shortens the migration times slightly, although the capillary cartridge temperature was still fixed at 20 °C. Additionally, the capillary was changed by a new one.

## 5. Validation of the Method

Among three bile salts newly tested in this work, only STC of 30 mM and SDC of 4.0 mM provide baseline resolution for all the four PALO stereoisomers. Their suitability for the analysis of enantiomeric impurities in real PALO product was checked by a real sample PALO injection. Its nominal content of effective ingredient, PALO (3aS, 2S), is 50 μg∙mL^−1^.

**Figure 4:**
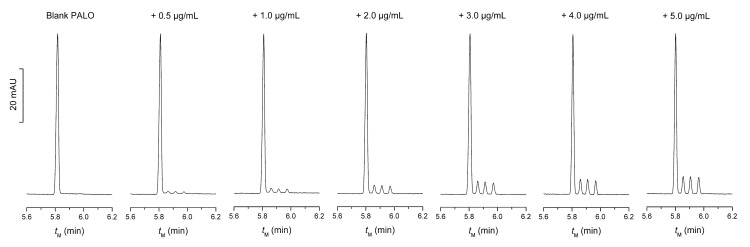
Electropherograms of PALO injection spiked with enantiomeric impurities to different concentrations. BGE composition is 30 mM STC in 30 mM sodium tetraborate of pH 9.2. Hydrodynamic injection at 10 kPa for 10 s. Detection wavelength: 254 nm. Other CE conditions are the same as in Figure 1. The four peaks from left to right are in the order of PALO (3aS, 2S), PALO (3aR, 2R), PALO (3aS, 2R), and PALO (3aS, 2R).

Firstly, the applicability of 30 mM STC was checked. As the enantiomeric impurities, i.e., PALO (3aR, 2R), PALO (3aS, 2R), and PALO (3aR, 2S), were not detected, the standards of them were spiked into the PALO injection to concentrations ranging from 0.5 to 5.0 μg∙mL^−1^. The spiked samples were injected directly. In order to improve the sensitivity and precision, a relatively long injection time of 10 s was used. As shown in Figure 4, the complete separation of all the stereoisomers can be achieved. Good precision, recovery, and a linear relationship between the peak areas and the impurity concentrations were obtained (Table 2). The limit of detection (LOD, S/N = 3) and limit of quantification (LOQ, S/N = 10) for the enantiomeric impurities are approximately 0.1 μg∙mL^−1^ and 0.3 μg∙mL^−1^, respectively. They are 0.2% and 0.6% percent, respectively, of the effective ingredient, PALO (3aS, 2S). Considering enough resolution between the peaks of the effective ingredient and adjacent impurity, it appears reasonable to assume that the analysis in the presence of even higher level of effective ingredient is also possible. Additionally, even lower concentration of impurities can be detected if a more sensitive detector other than UV detector was employed.

Although similar results have already been obtained in our previous works by using bile salt SC as a chiral selector, some approaches, including the addition of methanol [37] or additional surfactant (SDS) [38], had to be undertaken in order to accomplish complete separation of four stereoisomers. Compared to the reported methods based on SC, the method by using STC developed in this work has a distinct advantage of not using organic solvents or additional surfactant. Additionally, it provides good resolution of four stereoisomers in a wide range around the original pH of borate buffer; the adjustment and precise control of the pH are not needed. Furthermore, it consumes a little shorter separation time but provides better resolution between the peaks of the effective ingredient and adjacent impurity over those based on SC [37,38] in the analysis of real samples.

The robustness of the method was also checked (Figure S4). It was found that baseline resolution of four stereoisomers can be obtained in wide range of conditions, i.e., the applied voltage of 15 to 25 kV, the capillary temperature of 20 to 25 °C, the buffer concentration of 25 to 30 mM and the STC surfactant concentration of 25 to 50 mM, besides the resolution basically does not change in the range of pH 8.8 to 9.2 (Figure 1, STC). This demonstrated good robustness of the MEKC method for the separation of PALO stereoisomers by using STC as the surfactant and chiral selector.

Here, a different trend appears, compared to important effects on separation by the variation of conditions shown in Figure 1, Figure 2 and Figure 3. However, it can also be explained according to the principle of separation. As discussed above, the separation of charged solutes in MEKC is based on two effects: the mobility difference of free solutes in aqueous phase and the selectivity of micelles (retention difference). They can be synergistic or conflict with each other in the separation of diastereomers. In our opinion, if the two effects are in conflict and have similar strength in a certain range, small variation of conditions, particularly the pH or bile salt concentration, would have important effects on the separation because of tilting the balance of two effects. This explains the evident changes in separation at different conditions, as shown in Figure 1, Figure 2 and Figure 3. However, if two effects are synergistic or even in conflict but with a big difference in strength, variation of conditions in a certain range would not impact the separation obviously. So, good robustness of the method can be achieved under appropriate conditions, such as the use of 30 mM STC.

Finally, the suitability of 4.0 mM SDC for the analysis of spiked PALO injection was also tested. Unfortunately, the peaks of the effective ingredient and enantiomeric impurities cannot be resolved because of low sample capacity (data not given). Its application in real PALO products may be reliant on the use of other high sensitivity detectors, such as mass spectra or fluorescence.

## 6. Conclusions

All the four bile salts tested provide chiral recognition, as well as achiral selectivity, to some extent for PALO stereoisomers. SC and STC, as well as SDC and STDC, have the similar retention and selectivity but the separation parameters of SDC and STDC are rather different from those of SC and STC, suggesting that the structure of steroidal ring of bile salts has a greater impact on their interaction with analytes than the structure of side chain. Compared to SC, relatively low enantioselectivity of STC just avoids the peak overlapping between two pairs of enantiomers and is also enough for the chiral separation in each pair. As a result, it provides the complete resolution of four stereoisomers at 30.0 mM. The method has good robustness and is suitable for the analysis of a small amount of enantiomeric impurities in the presence of a high concentration of an effective ingredient in a real sample. Too large retention factors of SDC and STDC are unfavorable for the separation of PALO stereoisomers. Properly lowering the retention by using low surfactant concentrations in BGE improves both the chiral separation in each pair of enantiomers and the separation of diastereomers between two pairs, and shortens the separation time simultaneously.

## Figures and Tables

**Table 1:** The separation parameters of four bile salts at pH 9.2 for PALO stereoisomers ^a^.

	SC	STC	SDC	STDC
*k*_3aS,2S_	1.971	1.720	11.090	16.235
*k*_3aR,2R_	2.113	1.795	12.469	18.244
*k*_3aS,2R_	2.004	1.770	10.718	17.086
*k*_3aR,2S_	2.186	1.850	11.692	17.632
*α*_3aR,2R/3aS,2S_	1.072	1.043	1.214	1.124
*α*_3aR,2S/3aS,2R_	1.091	1.045	1.091	1.032
*α*_3aR,2R/3aS,2R_	1.054	1.014	1.163	1.068
*α*_3aR,2S/3aS,2S_	1.109	1.075	1.054	1.086
*α*_3aR,2R/3aR,2S_	0.966	0.970	1.067	1.035
*α*_3aS,2R/3aS,2S_	1.017	1.029	0.966	1.052
*t*_0_/*t*_mc_ (min)	4.283/9.659	4.519/11.236	4.092/11.924	4.285/11.612

^a^ Compositions of micellar solutions and CE conditions are the same as in Figure 1.

**Table 2:** Quantitative parameters of the developed method by using 30 mM STC for the analysis of the PALO injection spiked with enantiomeric impurities ^a^.

	PALO (3aR, 2R)	PALO (3aS, 2R)	PALO (3aR, 2S)
Calibration range (μg∙mL^−1^)	0.5–5.0	0.5–5.0	0.5–5.0
Regression equation	*y = ax + b* *y*: peak area (mV∙s) *x*: concentration of enantiomeric impurities (μg∙mL^−1^)		
Slope, *a*	1.421	1.407	1.391
Intercept, *b*	0.008	−0.014	0.030
Correlation coefficient, *R*^2^	0.999	0.994	0.996
Limit of detection (LOD, μg∙mL^−1^)	0.09	0.10	0.10
Limit of quantification (LOQ, μg∙mL^−1^)	0.31	0.32	0.32
Recovery (%) ^b^	99.1	95.6	96.7
Repeatability (RSD, %) ^b^			
Intra-day ^c^	3.3	3.7	2.2
Inter-day ^d^	6.9	4.5	4.2

^a^ Compositions of micellar solutions and CE conditions are the same as in Figure 4. ^b^ Values at an enantiomeric impurity concentration of 1.0 μg∙mL^−1^. ^c^ RSD of peak areas for 5 successive replications. ^d^ RSD of peak areas for 15 replicates in successively three days, five replicates each day.

## Data Availability

Data regarding this article will be provided upon request.

## Supplementary Materials

The following supporting information can be downloaded at: https://www.mdpi.com/article/10.3390/molecules27165233/s1, molecular structures of studied bile salts (Figure S1) and palonosetron hydrochloride (Figure S2), as well as electrophegrams that presents the measurement of the mobility of micelles (Figure S3) and robustness of the method (Figure S4).

## Abbreviations

PALO: palonosetron hydrochloride; SC, sodium cholate; STC, sodium taurocholate; SDC, sodium deoxycholate; STDC, sodium taurodeoxycholate; MOS, mobility standard.
