# Supplementary material for: Comparison of the Performance of Different Bile Salts in Enantioselective Separation of Palonosetron Stereoisomers by Micellar Electrokinetic Chromatography
Source: Molecules. 2022 Aug 16;27(16):5233. doi: 10.3390/molecules27165233 (PMC9415088; doi:10.3390/molecules27165233)
Supplement: Supplementary file 1 [file molecules-27-05233-s001.zip › molecules-1833440-SM.pdf]

Supplementary Materials

Comparison of the Performance of Different Bile Salts in Enantioselective Separation of Palonosetron Stereoisomers by Micellar Electrokinetic Chromatography

Shaoqiang Hu [1,*], Tao Sun [1], Rui Li [2], Dongdong Zhang [2], Yonghua Zhang [1], Zhuo Yang [1], Ge Feng [1] and Xuming Guo [2,*]

1 Henan Key Laboratory of Function-Oriented Porous Materials, College of Chemistry and Chemical Engineering, Luoyang Normal University, Luoyang 471934, China

2 School of Chemical Engineering & Pharmaceutics, Henan University of Science and Technology, Luoyang 471003, China

* Correspondence: shaoqianghu@sina.com (S.H.); xumingguo@163.com (X.G.)

Contents

Figure S1. Molecular structures studied bile salts

Figure S2. Molecular structure of palonosetron hydrochloride (PALO 3aS, 2S)

Figure S3. Determination of the migration time of micelles and the time window

Figure S4. Electropherograms of PALO stereoisomers at different conditions

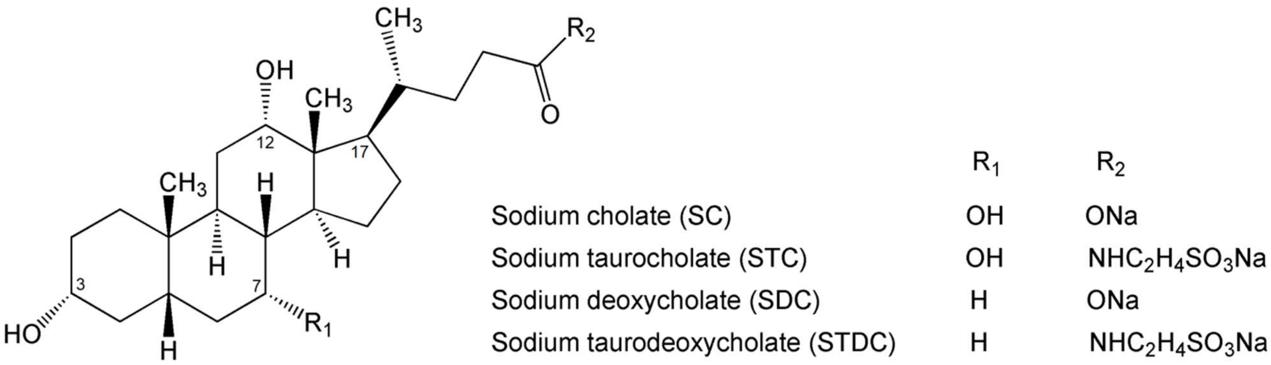

	R_1	R_2
Sodium cholate (SC)	OH	ONa
Sodium taurocholate (STC)	OH	$NHC_2H_4SO_3Na$
Sodium deoxycholate (SDC)	H	ONa
Sodium taurodeoxycholate (STDC)	H	$NHC_2H_4SO_3Na$

Figure S1. Molecular structures studied bile salts.

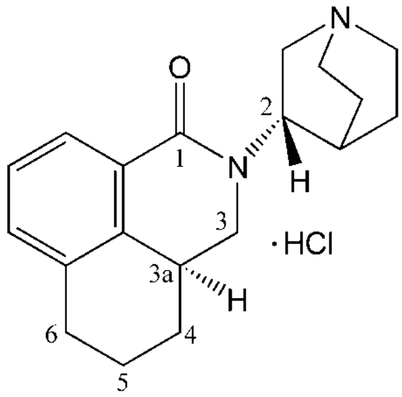

Figure S2. Molecular structure of palonosetron hydrochloride (PALO 3aS, 2S).

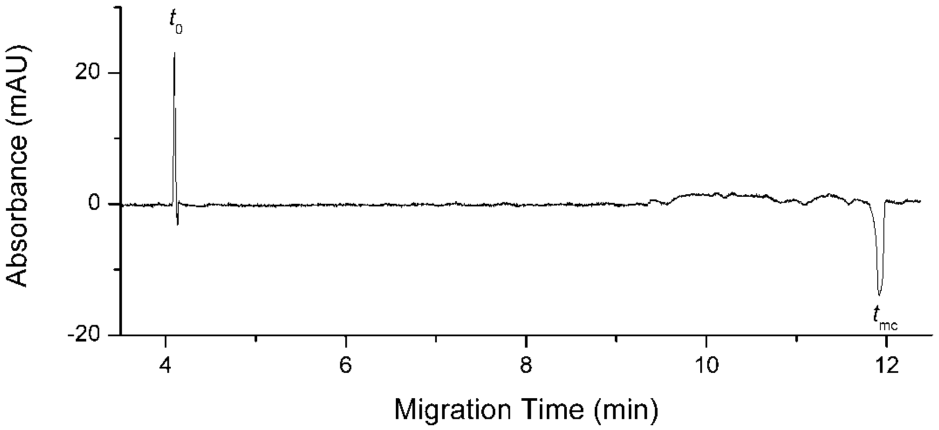

Figure S3. Determination of the migration time of micelles and the time window. Component of micellar solution (BGE) is 30 mM SDC in 30 mM sodium tetraborate buffer of pH 9.20. Capillary: id 50 μm, L_{tot} 60.0 cm, L_{eff} 50.0 cm. Capillary temperature: 20℃. Sample solution: 30 mM sodium tetraborate buffer of pH 9.20, containing 0.2 μL·mL^{-1} DMSO. Hydrodynamic injection at 10 kPa for 3 s. Detection wavelength: 205 nm. Applied voltage: 25 kV.

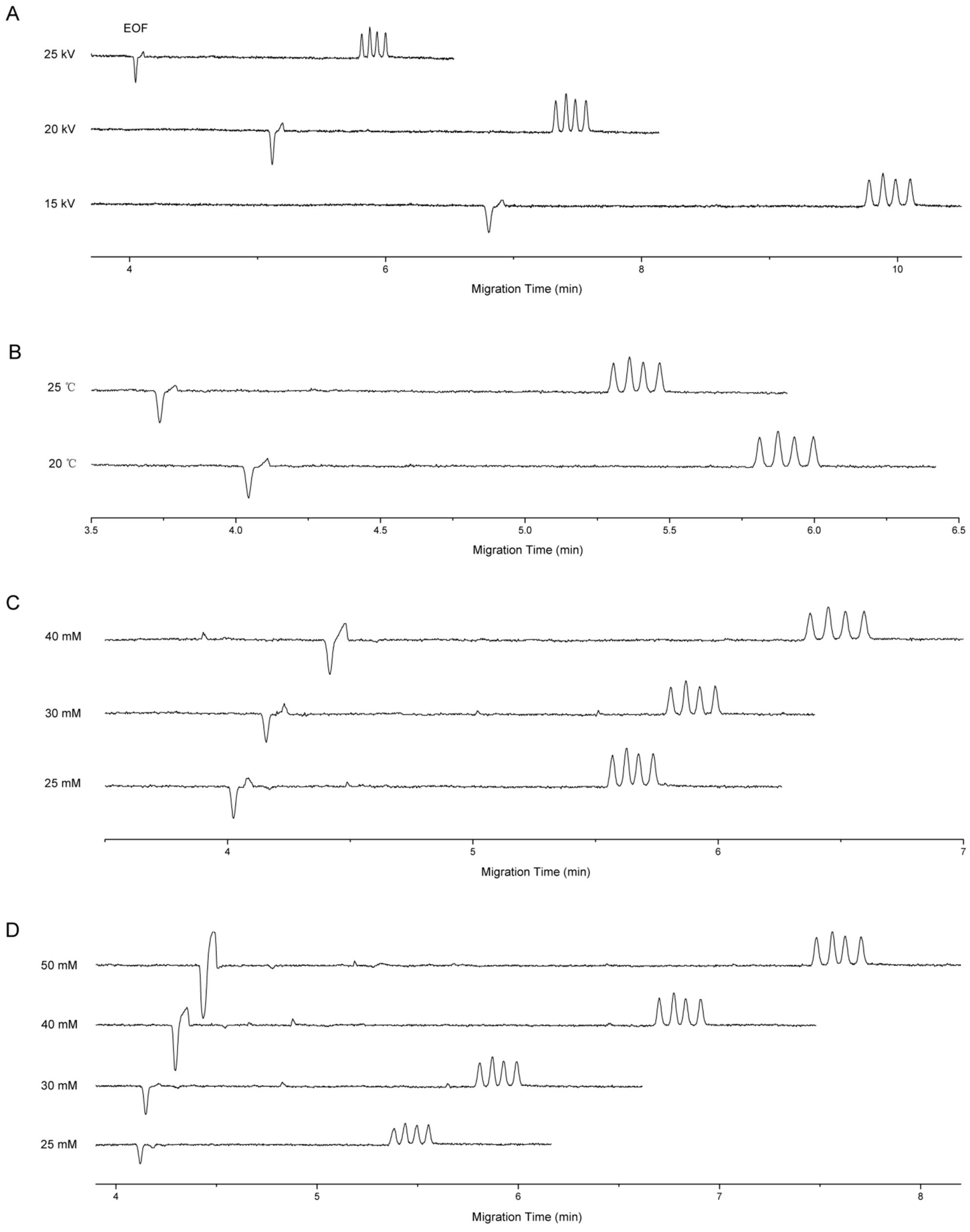

Figure S4. Electropherograms of PALO stereoisomers at different applied voltages (A), capillary temperatures (B), borate buffer concentrations (C) and STC surfactant

concentrations (D). Component BGE is 30 mM STC in 30 mM sodium tetraborate buffer of pH 9.20 except for specified. BGE Sample concentration: 0.1 $mg \cdot mL^{-1}$ for each stereoisomer. Hydrodynamic injection at 10 kPa for 1 s. Detection wavelength: 254 nm. Other CE conditions are the same as in Fig. S3 except for specified. The four peaks from left to right are in the order of PALO (3aS, 2S), PALO (3aR, 2R), PALO (3aS, 2R) to PALO (3aS, 2R) in all the electropherograms.
